# Changes of Oxidative Stress, Glutathione, and Its Dependent Antioxidant Enzyme Activities in Patients with Hepatocellular Carcinoma before and after Tumor Resection

**DOI:** 10.1371/journal.pone.0170016

**Published:** 2017-01-12

**Authors:** Shao-Bin Cheng, Hsiao-Tien Liu, Sin-Yuan Chen, Ping-Ting Lin, Chia-Yu Lai, Yi-Chia Huang

**Affiliations:** 1 Division of General Surgery, Department of Surgery, Taichung Veterans General Hospital, Taichung, Taiwan; 2 School of Medicine, Chung Shan Medical University, Taichung, Taiwan; 3 Graduate Program in Nutrition, Chung Shan Medical University, Taichung, Taiwan; 4 Department of Nutrition, Chung Shan Medical University, Taichung, Taiwan; 5 Department of Nutrition, Chung Shann Medical University Hospital, Taichung, Taiwan; Chang Gung Memorial Hospital Kaohsiung Branch, TAIWAN

## Abstract

The changes in and relationship between oxidative stress and the glutathione (GSH) antioxidant system in the plasma and tissues of patients with hepatocellular carcinoma (HCC) before and after tumor resection have not been clearly determined. We investigated the changes in oxidative stress, GSH status and its dependent antioxidant enzyme activities in HCC patients before and after tumor resection, and to determine the association of oxidative stress with GSH and its dependent antioxidant enzyme activities in plasma and tissues. This study employed a cross-sectional design. Forty-four men and 16 women with HCC were recruited. Fasting blood was drawn on the day before the tumor resection and one month after the tumor resection. HCC tissue and adjacent normal liver tissue were obtained at the time of surgical resection. Patients had significantly increased plasma malondialdehyde (MDA) and oxidized-low density lipoprotein levels but decreased GSH and oxidized GSH levels before tumor resection compared with the corresponding post-resection values. GSH and trolox equivalent antioxidant capacity (TEAC) levels and activities of GSH peroxidase were significantly increased while MDA level was significantly lower in HCC tissue when compared with the adjacent normal tissue. The pre-resection plasma MDA level was significantly correlated with pre-resection plasma GSH concentration, and MDA level in HCC and adjacent normal tissues. Pre-resection plasma GSH concentration was significantly correlated with GSH and TEAC level in HCC tissue. HCC patients had increased oxidative stress, decreased GSH, and lower dependent antioxidant capacities before tumor resection. However, hepatocellular tumor had increased GSH and TEAC levels as well as GSH peroxidase activities which might protect itself against increased oxidative stress.

## Introduction

Hepatocellular carcinoma (HCC) is the most common type of liver cancer and the mortality rate has risen over the past decade. Among risk factors related to HCC, increased oxidative stress and decreased antioxidant capacities have been suggested to be contributors to HCC [1–5]. Although the human body has different antioxidant systems to defend against the generation of free radicals, oxidative stress is elevated when antioxidant capacities are low, which disrupts the balance between pro- and anti-oxidants and further causes the oncogenesis and tumor progression of HCC [6,7].

Glutathione (GSH) is a thiol and tripeptide which is synthesized by glycine, cysteine, and glutamate in the liver and acts as a vital factor in metabolic protective functions, including the reduction of hydroperoxides, the quenching of free radicals, and the detoxification of xenobiotics [8]. The GSH-dependent antioxidant system consists of GSH and an array of functionally related enzymes, including glutathione S-transferase (GSH-ST), glutathione peroxidase (GSH-Px) and glutathione reductase (GSH-Rd). GSH-ST is not only capable of conjugating a number of potentially toxic electrophilic xenobiotics to the nucleophilic GSH, but can also catalyze reactions to reduce peroxides. GSH-Px can reduce hydroperoxides as well as H_2_O_2_ but also oxidizes GSH. GSH-Rd then reduces oxidized glutathione (GSSG) to the GSH.

The liver is the primary site for total body GSH turn-over and accounts for over 90% of GSH inflow into the systemic circulation [8]. Since the liver plays a central role in the interorgan GSH homeostasis, liver dysfunction might affect endogenous production and utilization of GSH, and possibly further lead to dysregulation of the GSH-dependent antioxidant system. Previous studies have shown that patients with liver cirrhosis or HCC had decreased plasma GSH concentration [9–11] and GSH-dependent antioxidant enzyme activities [2,5,12]. However, the changes in oxidative stress and the GSH-dependent antioxidant system after HCC tumor resection have not been reported. While a previous study indicated that plasma GSH concentration was highly correlated with the intrahepatic level [13], whether the expression of GSH and its dependent antioxidant enzyme activities in plasma are correlated with their expression in tissues during the development of HCC is unknown. In order to better understand the role of GSH and its dependent antioxidant enzymes in the development of HCC and after tumor resection, we investigated the changes in oxidative stress, GSH status and its dependent antioxidant enzyme activities in HCC patients before and after tumor resection, and determined the association of oxidative stress with GSH and its dependent antioxidant enzyme activities in plasma and tissues.

## Materials and Methods

### Study design and sample size calculation

This was a cross-sectional study of HCC patients’ pre- and post-resection oxidative stress, GSH status, and dependent antioxidant enzyme activities. Our group size was based on the requirement for the detection of a significant correlation coefficient of 0.4 between GSH in plasma and tumor tissue level with a power of 80% and a 2-sided test with an α of 0.05. The required sample size was at least 47 subjects. Our final recruitment number came to 60 HCC patients, which was greater than that determined in our original calculation.

### Patients

Patients were recruited from the division of general surgery of Taichung Veterans General Hospital, Taiwan, if they were confirmed to have primary HCC [International Classification of Diseases 9, code 155.0] and were scheduled to undergo tumor resection. Patients did not receive any treatment before surgery. Diagnosis and staging were confirmed by an oncologist and a pathologist based on the 7^th^ edition of the American Joint Committee on Cancer staging system [14]. Patients were excluded if they were younger than 20 y or older than 80 y, were pregnant or lactating, were receiving chemotherapy, or had a history of cardiovascular or renal diseases. Each subject signed off on the informed consent document. This study was approved by the Institutional Review Board of Taichung Veterans General Hospital (IRB TCVGH No. CF13197).

### Data collection and biochemical measurements

All subjects’ age, gender, smoking and drinking habits, use of medications and clinical outcomes (i.e., severity of liver disease according to the Child-Pugh classification, hepatitis, cirrhosis or ascites) were recorded. Subjects’ height and weight were measured and used to calculate their body mass index (BMI, kg/m^2^). Systolic and diastolic blood pressures (SBP and DBP) were measured after a resting period of at least 5 min.

Fasting blood samples were drawn and collected in vacutainer tubes (Becton Dickinson, Rutherford, NJ, USA) containing an anticoagulant or no anticoagulant on the day before the tumor resection (pre-resection) and one month after the tumor resection (post-resection). Blood specimens were taken to measure serum albumin, alanine (ALT) and aspartate (AST) aminotransferases, serum creatinine, blood urea nitrogen (BUN) and α-fetoprotein. Hepatocellular tumor tissue and adjacent normal liver tissue were obtained at the time of surgical resection. Histological examinations and grade of HCC were confirmed by a pathologist and classified as well-, moderately-, and poorly-differentiated HCC. Resected tissues were homogenized on ice in 1 mL phosphate saline buffer (pH = 7.4). Homogenates were centrifuged at 13,000 rpm for 10 min at 4°C. The supernatants were frozen at -80°C until analysis. Plasma and tissue samples were used to analyze oxidative stress indicators [i.e., malondialdehyde (MDA), oxidized-low density lipoprotein-cholesterol (ox-LDL)], GSH, GSSG, trolox equivalent antioxidant capacity (TEAC) and antioxidant enzyme activities (i.e., GSH-Px, GSH-Rd, and GSH-ST).

Plasma MDA was measured by thiobarbituric acid reactive substances at an excitation wavelength of 515 nm and an emission wavelength of 555 nm using a fluorescence spectrophotometer according to a method described by Lapenna et al. [15]. A commercial kit (Mercodia AB, Sylveniusgatan 8A, SE-754 50 Uppsala, Sweden) was used to measure plasma ox-LDL level. TEAC was measured according to a previous method [16]. GSH and GSSG were measured using GSH and GSSG commercial kits (BioVision Incorporate, Milpitas, CA, USA). Activities of GSH-Px, GSH-Rd, and GSH-ST were determined using GSH-Px, GSH-Rd, and GSH-ST commercial kits (Cayman Chemical Company, Ann Arbor, MI, USA), respectively. All analyses were performed in duplicate.

### Statistical analysis

The SAS statistical software package (version 9.3; Statistical Analysis System Institute Inc., Cary, NC, USA) was used for all data analyses. The Shapiro-Wilk test was performed to test the normal distribution. Demographic characteristics and biochemical data between the pre- and post-resection as well as between HCC tissue and adjacent normal tissue were compared using paired *t*-test or Wilcoxon signed rank test to determine any statistically significant differences. Chi-square or Fisher’s exact tests were used in the analysis of categorical variables. Partial Spearman correlation coefficients (*r*_*s*_) were used to analyze the associations of oxidative stress indicators with tumor grade, GSH, GSSG, GSH/GSSG ratio, TEAC, and GSH-dependent antioxidant enzyme activities in plasma and tissues after adjusting for age, gender, AST level, cirrhosis, and smoking and drinking habits. Statistical significance was defined as a two-sided *p* < 0.05.

## Results

Table 1 shows the demographic and health characteristics of HCC patients. All patients’ ages ranged from 30 to 79 years with a mean age of 59.2 ± 11.1 y. The severity of liver disease in 58 patients was Child-Pugh class A, and in 2 patients was Child-Pugh class B. Of the 60 patients, 82% of patients had a first-time diagnosis of HCC [new case] and 18% of patients had recurrent HCC. Twenty-eight patients were in cancer stage I and 32 patients were in cancer stage II, and more than two-thirds of patients had poorly differentiated HCC. The post-resection levels of BMI, AST, and α-fetoprotein in HCC patients significantly decreased, whereas BUN and creatinine significantly increased when compared with the corresponding pre-resection values. However, serum albumin, creatinine, and BUN levels were within normal range at pre- and post-resection.

**Table 1:** Demographic and health characteristics of patients with hepatocellular carcinoma^1^.

Parameters	Pre-resection [n = 60]	Post-resection [n = 60]
Age [y]	59.17 ± 11.10	
Male / Female	44 / 16	
BMI [kg/m^2^]	23.93 ± 3.24*	23.38 ± 3.41
Blood pressure		
SBP [mmHg]	128.58 ± 18.98	130.11 ± 18.46
DBP [mmHg]	76.68 ± 12.63	77.74 ± 13.29
Serum ALT [U/L]	63.57 ± 58.73	54.80 ± 48.05
Serum AST [U/L]	66.88 ± 62.71*	40.28 ± 36.56
BUN [mg/dL]	13.80 ± 5.07*	16.38 ± 6.72
Serum albumin [g/dL]	4.05 ± 0.37	4.19 ± 0.31
Serum creatinine [mg/dL]	0.82 ± 0.25*	0.91 ± 0.29
α-fetoprotein [μg/L]	2992.98 ± 11464.46*	304.36 ± 1326.88
Cases [n,%]		
New	49 [81.7%]	
Recurrent	11 [18.3%]	
Stage at diagnosis [n, %]		
Stage I	28 [46.7%]	
Stage II	32 [53.3%]	
Histological grading [n, %]		
Well-differentiated [I]	1 [1.7%]	
Moderately-differentiated [II]	13 [21.7%]	
Poorly-differentiated [III]	46 [76.7%]	
Cirrhosis [n, %]		
Yes	45 [75%]	
No	15 [25%]	
Ascites [n, %]		
Yes	2 [3.3%]	
No	58 [96.7%]	
Hepatitis [n, %]		
No hepatitis	4 [6.7%]	
Hepatitis B	38 [63.3%]	
Hepatitis C	17 [28.3%]	
Co-hepatitis B and C	1 [1.7%]	
Smoking habit [n, %]		
Yes	13 [21.7%]	
No	47 [78.3%]	
Drinking habit [n, %]		
Yes	6 [10%]	
No	54 [90%]	

^1^Values are means ± standard deviation. BMI, body mass index; SBP, systolic blood pressure

DBP, diastolic blood pressure; ALT, alanine aminotransferase; AST, aspartate aminotransferase; BUN, blood urea nitrogen.

*Values are significantly different from post-resection; *p* < 0.05.

Table 2 shows the values of oxidative stress indicators, GSH, GSSG, and dependent antioxidant enzyme activities in plasma and tissues. Patients had significantly lower levels of plasma GSH, GSSG and GSH-Rd activities but higher plasma MDA, ox-LDL, and GSH-ST activity values before resection compared with the corresponding post-resection values. For tissue levels (Table 3), the GSH and TEAC levels as well as activities of GSH-Px were significantly higher in HCC tissue when compared with the adjacent normal tissue. Meanwhile, the MDA level in HCC tissue was significantly lower than that of the adjacent normal tissue.

**Table 2:** Plasma reduced and oxidized, glutathione, indicators of oxidative stress, and antioxidant capacities in patients with hepatocellular carcinoma.

	Pre-resection [n = 60]	Post-resection [n = 60]
GSH [μmol/L]	38.86 ± 26.15*	48.66 ± 30.17
GSSG [μmol/L]	505.39 ± 98.24*	589.74 ± 119.92
GSH/GSSG ratio [× 10^−2^]	7.62 ± 4.36	8.24 ± 4.94
Oxidative stress indicators		
MDA [μmol/L]	1.01 ± 0.28*	0.97 ± 0.88
Oxidized-LDL [U/L]	37.64 ± 8.00*	33.72 ± 8.71
*Antioxidant capacities*		
TEAC [μmol/L]	4421.72 ± 616.52	4593.42 ± 496.29
GSH-Px [nmol/mL/min]	125.60 ± 85.79	148.84 ± 92.77
GSH-Rd [nmol/mL/min]	53.42 ± 13.50*	66.99 ± 15.32
GSH-ST [nmol/mL/min]	47.65 ± 22.51*	38.85 ± 16.27

Values are means ± standard deviation. HCC, hepatocellular carcinoma; GSH, reduced glutathione; GSSG, oxidized glutathione; MDA, malondialdehyde; Oxidized-LDL, oxidized-low-density lipoprotein cholesterol; TEAC, trolox equivalent antioxidant capacity; GSH-Px, glutathione peroxidase; GSH-Rd, glutathione reductase; GSH-ST, glutathione *S*-transferase.

*Values are significantly from post-resection; *p* < 0.05.

**Table 3:** Reduced glutathione, oxidative stress indicator, and antioxidant capacities in hepatocellular carcinoma tissues and adjacent normal tissues.

	HCC tissue	Adjacent normal tissue
GSH [μmol/g protein]	42.76 ± 20.59*	29.17 ± 14.92
Oxidative stress indicators		
MDA [μmol/g protein]	0.46 ± 0.50*	0.85 ± 0.42
*Antioxidant capacities*		
TEAC [μmol/g protein]	256.84 ± 82.76*	201.29 ± 58.38
GSH-Px [nmol/min/mg protein]	41.43 ± 17.10*	31.07 ± 10.95
GSH-Rd [nmol/min/mg protein]	61.05 ± 35.73	55.23 ± 94.66
GSH-ST [nmol/min/mg protein]	60.61 ± 75.51	70.94 ± 37.97

Values are means ± standard deviation. HCC, hepatocellular carcinoma; GSH, reduced glutathione; MDA, malondialdehyde; TEAC, trolox equivalent antioxidant capacity; GSH-Px, glutathione peroxidase; GSH-Rd, glutathione reductase; GSH-ST, glutathione *S*-transferase.

*Values are significantly different from adjacent normal liver tissue; *p* < 0.05.

The result of partial Spearman correlation coefficient showed no association between tumor histological grading and tissue GSH and oxidative stress (data not shown). Table 4 shows significant partial correlation coefficients of oxidative stress with GSH, GSSG, and antioxidant capacities. The pre-resection plasma MDA level was significantly correlated with the pre-resection plasma GSH, pre-resection GSH/GSSG ratio, MDA level in HCC tissue adjacent normal tissue after adjusting for potential confounders. Pre-resection plasma ox-LDL level was significantly associated with pre-resection plasma GSH and GSSG levels. Pre-resection plasma GSH concentration was significantly associated with GSH level in HCC tissue and TEAC level in HCC tissue after adjusting for potential confounders. However, post-resection plasma MDA or ox-LDL had no significant association with post-resection plasma GSH, GSSG, GSH/GSSG ratio and GSH-dependent antioxidant enzyme activities following adjustment for potential confounders (data not shown).

**Table 4:** Partial Spearman’s correlation coefficients [*r*_*s*_] of oxidative stress with glutathione, oxidized glutathione, and antioxidant capacities in plasma and tissues.

	Pre-resection plasma MDA [μmol/L]	Pre-resection plasma oxidized-LDL [U/L]	Pre-resection plasma GSH [μmol/L]
	*partial r*_*s*_^1^		
MDA [μmol/g protein]			
HCC tissue	0.64*	NS	NS
Adjacent normal tissue	0.47**	NS	NS
GSH [μmol/L]			
Pre-resection	0.35*	0.33*	–^2^
GSH [μmol/g protein]			
HCC tissue	NS	NS	0.57*
GSSG [μmol/L]			
Pre-resection	NS	0.34*	–
GSH/GSSG/ ratio			
Pre-resection	0.34*	NS	–
TEAC [μmol/g protein]			
HCC tissue	NS	NS	0.63*

^1^Values are partial correlation coefficient [*r*_s_]. Adjusting for age, gender, aspartate aminotransferase level, cirrhosis, smoking and drinking habits.

*p < 0.05

**p < 0.01.

HCC, hepatocellular carcinoma; MDA, malondialdehyde; Oxidized-LDL, oxidized-low-density lipoprotein cholesterol; GSH, reduced glutathione; GSSG, oxidized glutathione; TEAC, trolox equivalent antioxidant capacity; NS, non-significance.

^2^Not test.

## Discussion

Carcinogenesis may progress or be enhanced if an imbalance between oxidative stress and antioxidant defense capacities occur. Our patients had increased oxidative stress (i.e., MDA and ox-LDL levels) and decreased GSH and GSSG levels before they underwent HCC resection; however, the expression of oxidative stress and GSH status was reversed after the HCC resection. This phenomenon supports the notion that increased oxidative stress is an important pathogenic mechanism of HCC [2,3,5], and suggests that GSH might be utilized and oxidized to GSSG in order to cope with increased oxidative stress during the development of HCC. A significant decrease in plasma GSH among HCC patients was also observed in a previous study [3]. In contrast to previous results indicating a decrease of GSH might be due to the increase in plasma GSSG level and GSH/GSSG ratio in HCC patients [3,17], the pre-resection plasma GSH/GSSG ratio in our HCC patients actually remained steady when compared to the post-resection level. Even though our HCC patients were under increased oxidative stress status and GSH-Rd activity was decreased before tumor resection, their GSH-Rd activities were not been fully exhausted so GSSG could still be reduced to GSH, thereby preventing the accumulation of GSSG. That is to say, the balance of GSH/GSSG redox could be maintained and was not completely shifted to the oxidized state in our HCC patients. In the antioxidant defense system, GSH-Px is considered to be the primary antioxidant enzyme, while GSH-ST serves as the secondary antioxidant enzyme. Our results revealed that the GSH-Px activity only slightly but not significantly decreased and GSH-ST activity was significantly increased before HCC resection, which implies that our patients tolerated the oxidative stress well. However, it is not known whether these properties of GSH, GSSG, and GSH-dependent antioxidant enzyme activities would persist if carcinogenesis progressed for a longer period.

In contrast to the expression of MDA and GSH in the circulation, we detected significantly lower MDA and higher GSH, TEAC, and GSH-Px activity in HCC tissue when compared to adjacent normal tissue. Similar findings were also observed in rat hepatoma tissues [18] and human HCC tissue [19,20] as compared to non-HCC tissues. High intracellular GSH levels could protect cell death and promote tumor growth while a depleted intracellular GSH level might cause the onset of apoptosis and cytotoxicity [21–24]. Even though large amounts of free radicals are generated by tumor cells during carcinogenesis, tumors could protect themselves against increased oxidative stress through the elevation of intracellular GSH concentration. In addition to the overproduction of GSH that has been reported in breast or ovarian tumors [25–27], we also observed increased GSH concentration in hepatocellular tumor. The elevation of GSH level in HCC tissue might be contributed to the redistribution of GSH from plasma to HCC tissues since we observed a significant association between the GSH level in plasma and in HCC tissue. Although we did not evaluate HCC perfusion and tumor vascularization in the present study, primary HCC is known to be a typically and highly vascular malignant tumor through angiogenesis of hepatic artery and liver perfusion [28]. Thus, we speculate that hepatocellular tumor might be superior to its adjacent normal tissue in its ability to obtain GSH from plasma and decrease oxidative stress in order to facilitate tumor growth and invasion. In addition to the association between oxidative stress and GSH, the ratio of reduced and oxidized forms of GSH (GSH/GSSG) was used to critically evaluate oxidative stress in the cells [29]. The shifting of GSH/GSSG redox toward the oxidizing state would reduce cell proliferation and increase apoptosis [30]. Although we did not measure tissue GSSG level, the balance of GSH/GSSG redox seemed to be maintained in HCC tissue.

It worth noting that our results on the expression of oxidative stress, GSH status and its dependent enzymes in HCC tissue and adjacent normal tissue differ from those reported in the literature. In contrast to the findings of the present and previous studies [19,20], other results found an increase of MDA and a decrease of GSH levels in human HCC tissue compared to healthy or benign liver tissue [1,11,25,31]. Although we agree with the authors of previous studies who have suggested that reduced synthesis of GSH induced by liver dysfunction and increased oxidative stress might cause HCC tissue to lose its capacity to provide protection against oxidative impairment [1,11,25,31], this cannot explain why our HCC patients had lower MDA and higher GSH levels in HCC tissue when compared to its adjacent normal liver tissue. Yang et al. [7] reported that well-differentiated HCC cell lines expressed much higher GSH-Rd enzyme activities than poorly differentiated HCC cell lines. Lee et al. [11] indicated that most poorly differentiated HCC had the lowest levels of GSH, GSSG and GSH/GSSG ratio when compared to well- and moderately-differentiated HCC tissues. More than two-thirds of the patients we recruited had poorly differentiated HCC while only one patient had well-differentiated HCC. In addition, no HCC patients were in stage III or IV based on the TNM system. Therefore, sample size disequilibrium of each differentiated group and degree of HCC stage might be possible reasons for us not detecting any significant associations of tumor grade with oxidative stress or GSH level. The factors underlying the discrepancy in the expression of oxidative stress and GSH status in HCC tissue reported among different studies requires further investigation.

The strength of this study was that most of the patients were new cases, so this effectively eliminated the potential confounding effects of changes or modifications that occur in patients who were diagnosed a relatively long time ago. The second strength was that our sample size was 2.5 ~ 4.6 times larger than those analyzed in previous studies [1,11,25,31], which allowed more powerful statistical inferences. Another strength was that this was the first study to assess the changes of oxidative stress, GSH status, and its dependent antioxidant capacities before and after hepatocellular tumor resection. However, there were some limitations in this study. We could not obtain an adequate size of HCC and adjacent normal tissues in some patients, so some tissue parameters [i.e., GSSG and ox-LDL] could not be measured, which may have decreased the statistical power to observe the association between the level in plasma and tissues. The second limitation was possibly the timing for the post-resection and pre-resection evaluations, which may have been too close together. The post-resection data might therefore not be truly representative of the characteristics that would be observed under tumor-free conditions. The third possible limitation was that dietary intake was not recorded; it is not known that diet may influence oxidative stress status.

## Conclusions

The data herein indicate that although HCC patients had increased oxidative stress and decreased GSH-dependent antioxidant capacity in the circulation before tumor resection, hepatocellular tumor had increased GSH and TEAC levels as well as activities of GSH-Px which might protect itself against increased oxidative stress. It would be clinically valuable to know how hepatocellular tumors obtain more GSH from the circulation than adjacent normal tissue since there is currently no effective medical therapy for HCC.
